# Risk of SARS-CoV-2 Reinfections Among Healthcare Workers of Four Large University Hospitals in Northern Italy: Results of an Online Survey Within the ORCHESTRA Project

**DOI:** 10.3390/vaccines13080815

**Published:** 2025-07-31

**Authors:** Filippo Liviero, Anna Volpin, Patrizia Furlan, Silvia Cocchio, Vincenzo Baldo, Sofia Pavanello, Angelo Moretto, Fabriziomaria Gobba, Alberto Modenese, Marcella Mauro, Francesca Larese Filon, Angela Carta, Maria Grazia Lourdes Monaco, Gianluca Spiteri, Stefano Porru, Maria Luisa Scapellato

**Affiliations:** 1 Department of Cardiac, Thoracic, and Vascular Sciences and Public Health, University of Padova, Via Giustiniani 2, 35128 Padova, Italymarialuisa.scapellato@unipd.it (M.L.S.); 2 Occupational Medicine Unit, University Hospital of Padova, Via Giustiniani 2, 35128 Padova, Italy; 3 Preventive Medicine and Risk Assessment Unit, Via Giustiniani 2, 35128 Padova, Italy; 4 Department of Biomedical, Metabolic and Neural Sciences, University of Modena and Reggio-Emilia, 41125 Modena, Italy; 5 Occupational Medicine Unit, Department of Medical, Surgical & Health Sciences, University of Trieste, 34129 Trieste, Italy; 6 Occupational Medicine Unit, University Health Agency Giuliano-Isontina (ASUGI), 34129 Trieste, Italy; 7 Department of Diagnostics and Public Health, Section of Occupational Medicine, University of Verona, 37134 Verona, Italy; 8 Occupational Medicine Unit, Verona University Hospital, 37134 Verona, Italy

**Keywords:** COVID-19, multiple infections, healthcare personnel, multicentre study, comorbidities, symptoms

## Abstract

**Background/Objectives:** This retrospective multicenter study, conducted within the ORCHESTRA Project, investigated SARS-CoV-2 reinfections among 5777 healthcare workers (HCWs) from four University Hospitals (Modena, Verona, Padova and Trieste) in northern Italy, aiming to assess the risk of reinfection and its determinants, comparing the clinical characteristics of reinfections with those of first infections, and examining the impact of preventive measures and vaccination strategies. **Methods:** HCWs completed an online questionnaire between June and August 2022. The survey collected demographic, occupational, and clinical data, including information on first infections and reinfections. Statistical analyses were performed using SPSS 28.0, through bivariate and multivariate approaches. **Results:** Response rates were 41.8% for Modena, 39.5% for Verona, 17.9% for Padova, and 17.4% for Trieste. Among the respondents, 4.8% (*n* = 276) experienced 2 infections and 0.5% (*n* = 27) reported 3 infections, out of a total of 330 reinfection cases. Additionally, 43.0% (*n* = 2787) reported only one infection, while 51.5% were never infected. Reinfection rates increased across five study phases (based on the epidemiological context), likely due to the emergence of new SARS-CoV-2 variants. A booster vaccine dose significantly reduced reinfection risk. Higher reinfection risk was found among HCWs aged ≤30 years, those with chronic respiratory diseases, and those working in COVID-19 wards, particularly nurses and allied health professionals. Reinfections were associated with a lower frequency of symptoms both during the period of swab positivity and after a negative swab, as well as with a shorter duration of swab positivity. No significant differences in symptom duration were found between first infections and reinfections. **Conclusions:** Despite its limitations, the online questionnaire proved a useful tool. Natural infection and vaccination reduced both reinfection risk and symptom severity. Prior infections should be considered in planning vaccination schedules and prioritizing HCWs.

## 1. Introduction

The global emergency caused by the COVID-19 pandemic, triggered by the “severe acute respiratory syndrome coronavirus 2” (SARS-CoV-2), has posed significant challenges to the worldwide healthcare system, with a substantial impact on healthcare workers (HCWs). Being on the front lines in managing and treating patients affected by COVID-19, HCWs have been exposed to a high risk of infection [1,2,3,4]. Despite the widespread implementation of preventive measures and vaccination campaigns, SARS-CoV-2 reinfections have emerged as a growing concern, raising questions about the duration and effectiveness of post-infection and post-vaccination immunity. Reinfections represent a complex phenomenon influenced by various factors, including the surge of viral variants, individual immune responses, and the working conditions of HCWs.

Early studies focused on the incidence and determinants of SARS-CoV-2 breakthrough infections (BIs) among HCWs [5,6], typically defined as a positive SARS-CoV-2 test, obtained either by reverse transcription–polymerase chain reaction (RT-PCR) or a rapid antigen test, occurring more than 14 days after the final dose of the recommended vaccination regimen [7]. Subsequent research [8,9] found that previous SARS-CoV-2 infection and the standardized antibody titer were inversely related to the risk of BI. In particular, individuals with chronic diseases such as hypertension or cardiovascular diseases may have a lower serological response to SARS-CoV-2 vaccines [10] and thus an increased risk of BI. More recent data, collected during the Omicron surge, showed that vaccine effectiveness significantly wanes in the presence of new variants, particularly due to mutations in the spike protein, [11] thereby facilitating BI. Early investigations into SARS-CoV-2 reinfections were often limited to single-centre studies or meta-analyses aggregating monocentric data [12,13,14]. For example, studies conducted in France [15] and in Canada [16] reported no or rare reinfections among HCWs, during early follow-ups. However, with the emergence of the Omicron variant, reinfection rates among HCWs increased significantly, as demonstrated in some single-centre studies [17,18]. Multicentre studies are crucial for providing robust and generalizable data on SARS-CoV-2 reinfections as they reduce bias, improve statistical power, and allow comparisons across diverse populations and regions. To the best of our knowledge, there are no multicentre studies on SARS-CoV-2 reinfections among HCWs in Italy, particularly those covering multiple regions within the country. Available data often stem from social and geographical contexts that differ markedly from those in Italy. Moreover, while some studies included multiple hospitals within the same healthcare organization or region, often, their primary objective was not to investigate SARS-CoV-2 reinfections [9,17]. For instance, a study [17] from all India Institute of Medical Sciences, New Delhi, reported that 10.5% of HCWs faced reinfections, with a higher incidence among younger individuals and nurses. These results emphasize the Omicron variant’s immune evasion capabilities, which significantly reduced the protection conferred by prior infections and vaccinations, as documented by a marked increase in reinfection rates during this period [19].

One of the major challenges in assessing factors influencing SARS-CoV-2 reinfections has been the lack of comprehensive data, which is largely due to the difficulties in maintaining adequate phone tracking of infected HCWs during the most critical phases of the pandemic. Online health survey tools have proven valuable for real-time data collection during the COVID-19 pandemic [20]. They have allowed for rapid, cost-effective, and wide-reaching data acquisition, facilitating insights into virus spread, public health behaviours, and intervention effectiveness. Despite their usefulness, these tools require careful design to minimize biases and ensure data representativeness. This study aimed to evaluate the incidence and key factors influencing SARS-CoV-2 reinfections among HCWs employed in four major University Hospitals in northern Italy. Using data collected through a tailored online questionnaire as part of the ORCHESTRA Project, the analysis focused on assessing the risk of reinfection and its determinants, comparing the clinical characteristics of reinfections with those of first infections, and examining the impact of preventive measures and vaccination strategies.

## 2. Materials and Methods

### 2.1. Study Design, and Sample and Data Collection

A retrospective cohort study was conducted among HCWs from the University Hospitals of Verona, Padova, Trieste, and Modena, all located in northern Italy. The study included HCWs previously enrolled in the Horizon 2020 ORCHESTRA Project (https://orchestra-cohort.eu/) who completed an ad hoc online questionnaire between 1 June 2022 and 31 August 2022 and provided informed consent. These HCWs underwent regular SARS-CoV-2 screening with nasopharyngeal or salivary RT-PCR swab tests, as part of occupational health surveillance programmes implemented during the pandemic. This systematic testing protocol allowed for the timely detection of infections and reinfections, including asymptomatic cases. The frequency and strategy of screening varied over time, according to hospital-specific protocols shaped by epidemiological conditions and national and local recommendations. In addition, HCWs were also tested in cases of close contact with a confirmed positive individual or in the presence of clusters. This proactive surveillance approach was particularly relevant during the early phases of the pandemic, enhancing the detection of asymptomatic infections. The date of the first negative RT-PCR swab was recorded and used to estimate the duration of positivity. Screening and, in the case of infection, return-to-work after testing negative followed national and regional public health guidance, which changed over time based on the epidemic phase and differed depending on whether the individual was symptomatic or asymptomatic. Designed as an in-depth anamnesis tool, the questionnaire was sent by email to all eligible HCWs employed at the University Hospitals of Verona (*n* = 8183), Padova (*n* = 6728), Trieste (*n* = 6230), and Modena (*n* = 610). The survey collected data covering the period from February 2020 to August 2022 and included the following sections:

(a) Socio-demographic profile (e.g., country of birth, ethnicity, marital status, and educational level), for which the participants were asked to report the type of hospital setting in which they had primarily worked during the pandemic—namely, COVID-19 wards, referring to (low-, sub-, or high-intensity) inpatient care wards, and COVID-19 outpatient clinics.

(b) Smoking habits, which were self-reported and categorized as current smoker, former smoker, or never smoker. These categories referred exclusively to conventional cigarette use; no data were collected on cumulative exposure (e.g., pack-years) or on the use of electronic cigarettes or other nicotine delivery systems.

(c) Any SARS-CoV-2 infection (symptomatic or asymptomatic), including the date of a positive swab test, the time elapsed between one infection and another, eventual hospitalization, the length of hospital stay, and admission to an intensive care unit (ICU).

(d) Vaccination history, including vaccination dates, vaccine types, and the number of doses (0, 1, 2, 3, or 4) received prior to any SARS-CoV-2 infection.

(e) Symptoms developed during acute COVID-19 infection or after the first negative swab test, including the following: systemic [fever, chills, fatigue and/or malaise, muscle pain (myalgia) or joint pain (arthralgia), weight loss], gastrointestinal (abdominal pain, vomiting and/or nausea, loss of appetite and diarrhea), upper airway [changes in or loss of taste, loss of smell, runny nose (rhinorrhea), rhinitis] lower airway [shortness of breath (dyspnea), cough], pain (chest pain, headache, sore throat), ocular (conjunctivitis), skin (skin lesions), lymphatic and hemopoietic systems (lymphadenopathy, bleeding), neurological and mental (altered mental status and/or confusion, aphasia, memory loss, concentration disturbances, dizziness, seizures, inability to walk, fainting, insomnia, anxiety and depression), or any other symptoms.

(f) Symptoms persisting after the acute phase of the disease (i.e., after a negative swab test) for less than 15 days, 16–30 days, 31–60 days, or beyond 61 days.

(g) Symptoms newly developed after the first negative swab (including those listed above and others).

(h) Any pre-existing condition (defined as comorbidities with a medical diagnosis under treatment, grouped into broad disease categories as collected in the ORCHESTRA questionnaire), for which a binary term (yes/no) was created to account for the presence of any comorbidity if the responder was undergoing any therapy for the following diseases: diabetes, cardio-vascular; respiratory; liver; neurological; rheumatologic; immunosuppressive (sickle cell disease, splenectomy, congenital immune deficiency, and HIV).

In each section of the questionnaire, participants also had the opportunity to choose ‘other,’ ‘none,’ when indicated, or ‘unanswered,’ allowing for comprehensive data collection and flexibility in responses.

All infections were confirmed by RT-PCR tests (nasopharyngeal or salivary) for SARS-CoV-2. The date of the positive swab was used to determine the period of infection and the number of first infections, as well as first and second reinfections. Infections were classified as reinfections if they were confirmed via rt-PCR to have occurred for more than 90 days after a prior infection or if they were confirmed to have occurred during the first 90 days in the presence of epidemiological risk factors (i.e., significant exposure) with a resolution of clinical symptoms from the first episode and a negative RT-PCR swab before the new episode [21]. The data collected through the questionnaire, including missing data, were placed into a dataset and subsequently analyzed. Based on the epidemiological trend of the infections, the introduction of vaccination, and the emergence of the variant of concerns, five study phases were identified as follows: (I) 17 February 2020–19 July 2020, (II) 20 July 2020–31 January 2021, (III) 1 February 2021–31 October 2021, (IV) 1 November 2021–28 February 2022, and (V) 1 March 2022–1 August 2022. Although genomic sequencing data were not available, classification by study phase was used as a proxy for the predominant circulating SARS-CoV-2 variants.

The ORCHESTRA multicentre study was approved by the Italian Medicine Agency (AIFA) and the Ethics Committee of Italian National Institute of Infectious Diseases (INMI) Lazzaro Spallanzani (protocol code 436, date of approval 14 October 2021). Each centre also received approval from their local ethical committee.

### 2.2. Statistical Analysis

A descriptive analysis was conducted on HCWs’ demographic, occupational and clinical data, based on information collected through the ad hoc ORCHESTRA questionnaire. Data were presented as percentages for categorical variables or as means ± standard deviation (SD) for continuous variables. The continuous variables were compared using Student’s t-test, while categorical variables were compared using the Chi-square test. Missing information for each variable is indicated in the tables as “nr”, representing the number of subjects who did not respond to the specific question in the online questionnaire. To estimate the risk of infection (n° of infections ≥ 1) and reinfection (n° of infections ≥ 2), logistic regressions were performed, adjusting for age, sex, body mass index (BMI), smoking habits, job title, comorbidity, hospital ward and University Hospital (Verona, Padova, Trieste, and Modena). The ‘hospital wards’ variable was used as a proxy of occupational exposure, distinguishing between COVID-19 areas (e.g., inpatient wards or COVID-19 outpatient clinics) and non-COVID-19 areas. Logistic multivariate regression was used to evaluate the correlation between the frequency of reinfections, the hospital wards in which they occurred, the study phase and the vaccination status at the time of swab positivity. Adjusted odds ratios (adjORs) and 95% confidence intervals (95% CIs), were calculated. The outcomes of the first infections and the reinfections were compared using bivariate analysis, by assessing the onset of symptoms during infection and after a negative SARS-CoV-2 swab, ordinary hospitalization, hospitalization in ICU wards, the duration of positivity, and the duration of symptoms and hospitalization. Additionally, the frequencies of all symptoms among symptomatic infections were compared. To assess the frequency of symptomatic infections (those with at least one symptom) on the totality of the infections and to assess the frequency of the onset of the most frequent symptoms, comparing the likelihood of symptoms between first symptomatic infections and reinfections, logistic regressions were performed, adjusting for age, sex, comorbidity (yes/no), reinfection (yes/no), study phase and vaccination status upon positivity. AdjORs and 95%CIs were estimated. Additionally, linear multiple regressions were performed to investigate how the mean duration of positivity and the mean duration of symptoms varied according to the main covariates, such as age, sex, comorbidity (yes/no), reinfection (yes/no), study phase (from 1 to 5) and vaccination (yes/no). A *p*-value of <0.05 was considered statistically significant. Statistical analyses were performed using SPSS Statistics, version 28.0.

## 3. Results

During the study period, a total of 5777 HCWs filled in the questionnaire, with a response rate of 41.8% (255/610) for Modena, 39.5% (3234/8183) for Verona, 17.9% (1206/6728) for Padova and 17.4% (1082/6230) for Trieste. The characteristics of the study sample are described in Table 1. Just over two-thirds of the respondents (*n* = 4349) were female, with a mean age of 45.5 ± 12.1 years. Specifically, 15.8% were under 31 years old, 32.4% were between 31 and 49 years old and 35.3% were over 50 years old. Almost all respondents in the sample (94.8%) had Italian citizenship, and 78.1% were Caucasian. The majority of the study population was married (44.3%), followed by singles (30.3%), while smaller portions were cohabitants (12.9%) and divorced individuals (8.6%). Most participants had a higher education, with 27.8% holding a high school diploma, 40.8% holding a degree, and 21.6% having completed postgraduate education, whereas only a small proportion (5.9%) had only a secondary school diploma. The mean BMI was 24.0 ± 4.5. Regarding smoking status, most had never smoked (62.2%), 26.8% were current smokers, and a smaller group (9.6%) were former smokers. About a quarter of the respondents reported having at least one comorbidity, including diabetes (1.7%), cardiovascular diseases (9.4%), chronic respiratory diseases (3.5%), autoimmune/rheumatological diseases (6.6%) and immunosuppressive conditions (2.7%). As for the job titles, 33.5% of the respondents were nurses, 12.1% were physicians, 9.4% were residents, 9.5% were allied health professionals, 13.3% were classified under other health personnel (including pharmacists, obstetricians, psychologists, physiotherapists, students and health technicians), and 18.2% were classified as other non-health personnel (such as administrative and laboratory staff). During the study period, 19.9% of the respondents primarily served in COVID-19 hospital wards, with 11.4% of participants in low-intensity wards and 10.8% in high-intensity wards. Additionally, 2.2% worked in COVID-19 ambulatories. Almost half of the respondents (*n* = 2787, 48.2%) experienced at least one infection. Specifically, 43.0% (*n* = 2484) had 1 infection, 4.8% (*n* = 276) had 2 and 0.5% (*n* = 27) had 3, totalling 3117 infections (2787 first infections and 330 reinfections). A total of 303 HCWs experienced ≥2 reinfections. The time elapsed between reinfections was a mean value of 389 ± 223 days. In particular, 90 reinfections occurred less than a year apart, and 132 occurred more than a year later. Among the HCWs who responded to the questionnaire, 92.4% were vaccinated: 0.3% had received one dose, 4.9% had received two doses, 86.9% had received three doses, 0.3% had received four doses, and 0.4% were unvaccinated. It should be noted that 7.2% of respondents did not provide an answer to this question. Among the vaccinated participants, 97.4% (5201 out of 5338) received the BNT162b2 (Pfizer-BioNTech) vaccine. Due to this overwhelming predominance, further stratification by vaccine type or classification into homologous versus heterologous regimens was not performed, as it would not have yielded meaningful subgroup comparisons.

Table 2 shows the distribution of respondents with at least one infection (*n* = 2787) and those with reinfections (*n* = 303) according to the main covariates of interest. In total, 54.4% of nurses, 49.4% of allied health professionals, 47.9% of physicians, 58.5% of residents and 48.2% of those with at least one comorbidity reported at least one infection. Notably, 6.9% of nurses and allied health professionals, 4.0% of physicians, 5.9% of residents and 5.6% (77/1355) of those with at least one comorbidity reported a reinfection (Table 2). Multivariate analysis showed a significantly higher risk of reinfection among nurses and allied health professionals, compared to other non-health personnel, as well as among subjects with chronic respiratory diseases, HCWs who worked in COVID-19 hospital wards and those aged ≤ 30 years.

Table 3 shows the distribution of reinfections among all infections. The rates of reinfections contracted in the COVID-19 high-intensity hospital wards were significantly higher compared to the rates of those contracted out of the hospital wards. However, it should be noted that 9.3% and 11.9% of reinfections also occurred in non-COVID outpatient clinics and non-COVID-19 hospital wards, respectively, though these results are not statistically significant. With regard to the study phase and the variants in circulation, the multivariate analysis confirmed a significantly increasing frequency of reinfection from phase 2 onwards. On the other hand, with regards to the SARS-CoV-2 vaccine, the administration of the third dose contributed significantly to reducing the risk of reinfection.

Bivariate analysis comparing the outcomes of reinfections vs. first infections showed a significantly lower frequency of symptoms during reinfections, both while HCWs were positive and after they tested negative for SARS-CoV-2 (Table 4). Overall, 64.3% of infections were symptomatic (*n* = 2005), with a significantly higher frequency of symptoms in first infections, 66.6% (*n* = 1855), compared to reinfections, 45.5% (*n* = 150). Moreover, 44.7% of the respondents (*n* = 1394) reported symptoms after testing negative from a SARS-CoV-2 swab, with a significantly higher frequency among first infections of 46.5% (*n* = 1296) compared to reinfections, 29.7% (*n* = 98) (Table 4), as well. Furthermore, the mean duration of positivity and the mean duration of symptoms were significantly shorter in reinfections compared to first infections. However, there were no significant differences between first infections and reinfections in the duration of symptoms after a negative SARS-CoV-2 swab. Hospitalization occurred in 0.6% of cases (*n* = 19) and hospitalization in an ICU occurred in 0.1% of cases (*n* = 4), without significant differences between first infections and reinfections (Table 4).

The multivariate analysis, as shown in Table 5, Table 6 and Table 7, confirmed that reinfections were associated with a lower frequency of symptoms (both during positivity and after a negative SARS-CoV-2 swab) and a shorter duration of positivity. As shown in Table 5, symptomatic infections were significantly less frequent among the reinfections and among HCWs who had been administered the second dose of vaccine. Conversely, symptomatic infections were more frequent among females and younger subjects, as well as during the later study phase compared to the initial phase. Furthermore, the frequency of infections with persistent symptoms after a negative SARS-CoV-2 swab was significantly lower among reinfections, while it was significantly higher in those respondents who had at least one comorbidity. The linear regression analysis in Table 6 demonstrates that the mean duration of positivity increased with age and decreased across advancing study phases. Additionally, the duration was shorter among vaccinated HCWs and among those with reinfections compared to first infections. The mean duration of symptoms increased with age and the presence of at least one comorbidity. In contrast, the duration decreased with the number of vaccinations and as the study phases progressed. No significant difference in the duration of symptoms was found between first infections and reinfections (Table 7).

As reported in the Supplementary Materials (Tables S1–S5), among the 2005 symptomatic infections analyzed, the most frequent symptoms during positivity were fever (57.4%), fatigue and malaise (74.0%), myalgia–arthralgia (57.3%), cough (54.1%), and headache (52.3%). During reinfections, a number of symptoms, including those listed above, were less frequent compared to first infections. Specifically, fever, fatigue and malaise, myalgia–arthralgia, cough, headache, loss of taste, loss of smell, dyspnoea, lack of appetite and vomiting and nausea were significantly less frequent (Table S1). Regarding symptoms after a negative SARS-CoV-2 swab, the most frequent ones were fatigue and malaise (49.4%) and myalgia and arthralgia (22.7%). No significant differences in any symptoms were observed when comparing symptomatic first infections to symptomatic reinfections (Table S2). The multivariate analysis shown in Tables S3–S5 confirms that there was a lower frequency of symptoms among reinfections, with a particularly significant reduction in fever, fatigue and malaise, myalgia–arthralgia, cough, headache, loss of appetite, and loss of taste or smell. The frequency of fatigue/malaise and dyspnoea appears significantly higher in those with at least one comorbidity (Tables S3 and S4). Regarding study phases, there was a higher frequency of loss of taste or smell, dyspnoea, and loss of appetite, as well as a lower frequency of cough, in the initial study phases compared to the later ones. Regarding vaccination status at the time of positivity, the analyses did not indicate any relevant differences in the distribution of symptoms.

## 4. Discussion

This study is the first multicentric Italian investigation focusing on the SARS-CoV-2 reinfections in a relatively large sample of HCWs. In particular, it analyzed the incidence of SARS-CoV-2 infections from 17th February 2020 to 31st August 2022, the main determinants related to an increased risk of infection and reinfection and any differences in outcomes between first infections and reinfections among HCWs employed at the University Hospitals of Verona, Padova, Trieste, and Modena, who completed an online questionnaire between 1st June 2022 and 31st August 2022. The analysis revealed that 4.8% of respondents experienced two infections, and 0.5% reported three infections, while 43.0% had only one infection and 51.5% did not report SARS-CoV-2 infection at all. Overall, there were 2787 first infections and 330 cases of reinfection. A higher risk of reinfection was observed among HCWs employed in COVID-19 hospital wards, particularly nurses and allied health professionals, as well as those aged ≤ 30 years and those with chronic respiratory diseases. Additionally, reinfections increased from phase 2 to subsequent study phases. On the other hand, with regards to the SARS-CoV-2 vaccine, the administration of a third dose contributed significantly to a reduction in the risk of reinfection. Reinfections were characterized by a lower frequency of symptoms (both during positivity and after a negative SARS-CoV-2 swab) than that for the first infections. Reinfections also had a shorter duration of positivity, but not a shorter duration of symptoms.

The data collected through the online questionnaire align with those from a previous study conducted among 8029 HCWs at the University Hospital of Padova [22], confirming that younger HCWs, healthcare personnel, and unvaccinated individuals exhibited a higher risk of SARS-CoV-2 infection. This is consistent with a systematic review and meta-analysis of 54 studies that showed an increased risk of being positive in frontline HCWs [23]. Moreover, seroprevalence surveys conducted in Italy in the early phase of the pandemic, when there was a significant shortage of personal protective equipment and before the introduction of vaccination, found that HCWs had significantly higher antibody prevalence compared to other occupational groups or the general population, reflecting their increased occupational exposure risk [24,25]. Our results specifically highlight an increased risk of first infections among HCWs aged ≤ 30 years, consistent with other studies [17,26,27,28]; this is likely due to younger individuals having more intense social relationships and higher contact rates [29]. Moreover, a higher risk of infection was confirmed to be associated with chronic respiratory diseases [30], and a separate study [31] found that individuals with chronic kidney disease were more likely to test positive in the adjusted analyses.

Overall, 5.3% of HCWs who responded to the survey questionnaire experienced reinfections, a finding consistent with the literature data when considering the study period and the variants of concern. Notably, we observed a significant increase in the frequency of reinfections from phase 2 onward, with the majority of these reinfections occurring particularly in phases 3, 4, and 5 compared to the earlier phases. While we could not determine the viral variant for each infection due to the absence of genomic sequencing, temporal classification by study phase provided a meaningful epidemiological context. This allowed us to interpret trends in reinfection risk in relation to major shifts in variant circulation and potential immune escape dynamics. Early studies on the risk of SARS-CoV-2 reinfection among HCWs, published in 2020, reported no reinfection among 4168 HCWs in France between March and December 2020 [15] and indicated that reinfection remained a rare event among 569 unvaccinated Canadian HCWs with a primary infection [16] followed between 21st August 2020 and 1st March 2022. A meta-analysis of three studies evaluating the prevalence of SARS-CoV-2 reinfection among HCWs in Spain and the United Kingdom (*n* = 26,153) from their inception to 17th July 2021 found that the pooled prevalence of reinfection was 3% [32]. However, recent evidence suggests that the risk of reinfection could be significantly higher with the emergence of the Omicron variant compared with previous variants. Indeed, since November 2021, during the Omicron period, the proportion of reinfections increased exponentially [18,33,34,35]. For example, in a study of 11,474 HCWs from all India Institute of Medical Sciences in New Delhi, first-time or recurrent SARS-CoV-2 infection was reported by 22%, during the period of 01st December 2021- 25th February 2022. Among 3545 HCWs who had a previous SARS-CoV-2 infection, reinfection was seen in 1007 HCWs (28.4%) with a greater risk of reinfections observed in the Omicron surge compared to earlier pandemic periods [17]. Moreover, between 11 March 2020 and 28 February 2022, reinfection occurred in 9.5% of 2355 HCWs employed in a Turkish oncology hospital, predominantly during the Omicron variant period [35].

We observed a higher risk of reinfections among HCWs who worked in COVID-19 hospital wards, particularly for nurses and allied health professionals, as well as for those with chronic respiratory diseases and those aged ≤ 30 years. This finding aligns with the results of Malhotra et al.’s study [17], showing higher infection and reinfection rates among younger age groups compared to older individuals (≥45 years) during the Omicron wave. This trend is also consistent with their earlier findings, where fewer reinfections were observed in older age groups during earlier periods, likely due to the greater severity of first infections in these groups [36]. Similarly, another study reported that a significantly higher proportion of HCWs aged 18–30 years were infected with the Omicron variant compared to those aged 55 years and above [37]. De Maria et al. [38] also found that reinfections in a cohort of 6234 vaccinated HCWs at the University Hospital of Bari were more frequent among younger HCWs, particularly physicians.

Piazza et al. found that during the study period (September 2021–May 2022), HCWs were more than twice as likely to be reinfected compared to non-healthcare workers [34]. This finding is supported by other literature data which highlights the elevated risk of SARS-CoV-2 reinfection among front-line workers due to their high exposure to the virus [39,40,41,42,43,44,45] and among nurses. In contrast, in a study of 829 HCWs, Chivu et al. [46] observed that age and occupational category did not show statistically significant differences between those who were reinfected and those who were infected only once. It is noteworthy, however, that this finding stems from a single study with a limited sample size, which may constrain the generalizability of the results.

We observed a higher risk of reinfection specifically among HCWs with chronic respiratory diseases. Piazza et al. [34] reported that 18.28% of patients who experienced SARS-CoV-2 reinfection had one or more comorbidities. The most significant conditions associated with reinfection included respiratory diseases such as bronchopneumopathy, asthma, as well as respiratory failure requiring oxygen therapy, cardiovascular conditions like heart failure, and chronic renal failure, particularly in patients undergoing dialysis, among other comorbidities. A systematic review published in 2022, covering data from 1st December 2019 to 1st September 2021, also identified hypertension and obesity as the most common comorbidities among reinfected patients, followed by end-stage renal failure, asthma, chronic obstructive pulmonary disease (COPD), dementia, dyslipidaemia and type 2 diabetes [45]. Other studies have also further reported a higher risk of reinfection in individuals with end-stage renal failure, hypertension, diabetes, chronic respiratory disease, liver disease and a history of cardiovascular disease [47,48,49,50]. This could be due to an impaired immune response, which limits lasting immunity post-infection.

We found that the booster dose of SARS-CoV-2 vaccine contributed to significantly reducing the risk of reinfection, according to the existing literature, which consistently shows that the risk of reinfection decreases with an increasing number of COVID-19 vaccines doses [13,51,52]. A meta-analysis, which included data up to 31st July 2022, from over 18 million previously infected and recovered individuals, demonstrated that compared to natural immunity alone, vaccination reduced the likelihood of reinfection by approximately 50% [13]. However, vaccine effectiveness wanes significantly after new variants surge, making anti-S antibody titers an unreliable predictor for the optimal timing of subsequent booster doses. For instance, the cumulative incidence of breakthrough infections (BI) among vaccinated HCWs in the ORCHESTRA Project was significantly higher during the Omicron Variant surge compared to previous variants of concern [11]. Furthermore, evidence in the literature suggests a significant risk of reinfection among immunocompromised patients or those experiencing a decline in antibody levels over time [32]. In our HCWs population, the mean time between reinfections was approximately 13 months, indicating that immunity, acquired through both infection and vaccination, likely waned over time, as shown in previous studies [53,54,55]. The ORCHESTRA research group also demonstrated that higher anti-S levels at nine months post-vaccination were significantly associated with having received three doses of the vaccine and with prior SARS-CoV-2 infection, whereas lower levels were linked to older age, a longer interval since the last vaccine dose, and multimorbidity [10].

We found no significant differences in the risk of reinfections between sex. However, data in the literature are contrasting. Chivu et al., like us, found that sex did not have any significant impact on the risk of reinfections in HCWs before or during the Omicron dominance in Romania [46]. In contrast, some studies highlighted an increased risk of reinfection among female HCWs [17,18,29,38]. However, a systematic review and metanalysis of 25 observational studies (most of which were conducted in China) among COVID-19 patients found a higher overall estimate of reinfection among males compared to females [12].

Another significant finding of our study was that reinfections required hospitalization in only 0.6% of cases (*n* = 19) and ICU admission in 0.1% of cases (*n* = 4). This is in line with previously published results which highlight that vaccination reduces the severity of reinfections [56]. Persistent symptoms after a negative RT-PCR swab were significantly less frequent in reinfections. Conversely, booster vaccination (third dose) did not appear to reduce the likelihood of either symptomatic infection during positivity or persistent symptoms post-infection. Moreover, a previous natural infection appeared to be the most consistent protective factor across both outcomes. Reinfections were characterized by a lower frequency of symptoms (both during positivity and after testing negative for SARS-CoV-2 swab), compared to first infections, which were more likely to be symptomatic. Additionally, we found that reinfections had a shorter duration of positivity to the swab test compared to first infections. However, no significant difference in the duration of symptoms was found between first infections and reinfections. These observations are in line with global evidence that the Omicron variant is associated with a milder spectrum of COVID-19 disease, resulting in fewer hospitalizations, ICU admissions and shorter hospital stays compared to Delta variant and previous waves [17,18,57]. In addition, previous data from the ORCHESTRA Project showed that a first infection provides protection against asymptomatic reinfections and even more against symptomatic infections [58]. Similarly, the UK SIREN study reported that prior SARS-CoV-2 infection was associated with an 84% reduced risk of reinfection among healthcare workers, highlighting the long-term protective effect of natural immunity [9]. Consistently, a large meta-analysis [13] confirmed the long-lasting protective effect of natural immunity against both SARS-CoV-2 reinfection and hospitalization, based on data from over 15 million individuals. Our current findings reinforce this evidence by supporting the protective role of previous natural infection, particularly in reducing the clinical impact of reinfection episodes. Large-scale community-based comparisons across multiple Omicron waves regarding reinfection characteristics, risk factors, and the protection provided by previous infection and vaccination are limited. However, a recent work analyzing 45,000 reinfections from the UK’s national SARS-CoV-2 Infection Survey found that reinfections were associated with a lower viral load and a lower percentage of self-reporting symptoms compared to first infections [59].

Our analysis showed that symptoms in SARS-CoV-2 reinfections occurred with a significantly lower frequency compared to the first infection. In particular, fever, fatigue, myalgia/arthralgia, cough, headache, loss of taste and smell, dyspnoea, lack of appetite and vomiting and nausea were less common in reinfections. These findings are consistent with a recent systematic review and meta-analysis [60], which included 19 studies conducted on the general population, totalling 34,375 reinfections and 5,264,720 first infections, with data collected up to December 2022. This study highlighted a significant reduction in clinical severity of reinfections compared to first infections, with the risk of severe disease reduced by 86%. Additionally, 41.77% of reinfections were asymptomatic, while 51.83% presented mild symptoms. However, the authors did not provide a direct comparison of the frequency of these symptoms between first infections and reinfections. Our data partially diverge from the findings of another systematic review and meta-analysis [61], which included 23 studies conducted on the general population as well as on HCWs, students, and athletes, with data collected up to 31 March 2023, totalling 23,231 reinfections. This meta-analysis did not find significant differences in clinical presentation between the first infection and reinfection. According to this study, symptoms such as fever, cough, myalgia, fatigue, and headache occurred with similar frequency in both episodes. However, the authors highlighted high heterogeneity across studies, selection biases in the populations analyzed, and variability in the definition of reinfection, which may have masked potential differences in clinical presentation. Instead, in our study we applied a consistent approach in all centres which reduced the heterogeneity.

### Strengths and Limitations

This study is the first multicentric Italian investigation focusing on the impact of SARS-CoV-2 reinfections in a large sample of HCWs, with a detailed collection of demographics, occupational, and clinical data, including information on first infections and reinfections, and risk estimates adjusted for a high number of potential confounders such as comorbidities. In addition, data were gathered on COVID-19 symptoms and hospitalization. Moreover, the presence of systematic screening programmes across participating hospitals, particularly during the early phases of the pandemic, likely increased the likelihood of detecting asymptomatic infections and reinfections, thereby improving the sensitivity of our outcome estimates. The use of an online questionnaire provided advantages, including rapid data collection and wide outreach; however, it also presented limitations, such as potential recall bias regarding the timing of infections, vaccinations and symptomology, reporting bias in self-reported information, and a lack of objective clinical verification of infections and comorbidities. Despite these limitations, as well as missing data that could overestimate unvaccinated and uninfected HCWs, the findings align with the existing literature, supporting their reliability [13,51,59,60].

Additionally, the investigation was limited to the sub-sample of respondents, which may not necessarily be representative of the entire study cohort, potentially introducing selection bias. Nevertheless, among the 5777 respondents, approximately half had experienced at least one SARS-CoV-2 infection and half had not, indicating that participation was not disproportionately higher among those who had been infected. Moreover, the response rates varied across the participating health centres (ranging from 17% to 42%). Common response rates for online surveys among company employees range from 30 to 50%, with 50–60% being considered good and higher rates indicating strong engagement [62]. However, given the structural complexity and heterogeneity of the healthcare settings involved, applying a weighting system to underrepresented subgroups was not considered appropriate, as it could have introduced further bias into the analysis.

Lastly, genomic sequencing data were not available, preventing variant-level identification. However, stratification by pandemic phase was used as a proxy for the predominant variants and to assess their role in reinfections.

## 5. Conclusions

This study underscores the ongoing concerns about SARS-CoV-2 reinfections among HCWs, revealing significant and persistent risks despite extensive vaccination and preventive efforts. The findings show that reinfection risk is particularly high among younger HCWs, those working in COVID-19 wards, and individuals with chronic comorbidities. The study also revealed that reinfections are generally less symptomatic and last for a shorter duration compared to first infections, although their incidence has risen with the emergence of new virus variants, especially in the later phases of the pandemic.

These observations highlight the importance of continuous pandemic monitoring and the need to adapt infection control and vaccination strategies in response to emerging variants and evolving epidemiological evidence. While a third vaccine dose significantly reduces reinfection risk, it does not provide complete protection, particularly against newly circulating variants. In addition, our results suggest that previous natural infections and vaccination confer a protective effect against both reinfection and symptom severity. Therefore, an individual’s infection history as well as vaccination status should be considered in vaccination schedules and when prioritizing HCWs for immunization to ensure tailored and effective preventive strategies in the healthcare setting.

Online questionnaires have proven valuable for collecting detailed and standardized data on the pandemic’s impact among HCWs, offering a cost-effective and flexible approach. However, their use also presents challenges such as bias minimization and data representativeness. In fact, various forms of bias may arise. Selection bias can occur if certain groups of HCWs are more likely to participate, leading to a non-representative sample. Response, recall, and reporting biases may also affect the accuracy of self-reported data, especially when sensitive or retrospective information is involved. Despite these limitations, this method has the potential for supporting a more efficient acquisition of critical data in order to better understand the determinants of reinfections, which will be useful for developing evidence-based recommendations and thereby helping to mitigate reinfection risks and safeguard essential healthcare personnel.

## Figures and Tables

**Table 1:** Characteristics of the respondents to the questionnaire.

	Total Subjects *n* = 5777			Total Subjects *n* = 5777	
	*n*	(%)		*n*	(%)
**Sex**			**Job Title**		
Female	4349	(75.3)	Physicians	697	(12.1)
Male	1363	(23.6)	Residents	544	(9.4)
nr	65	(1.1)	Nurses	1937	(33.5)
**Age (mean ± SD)**	45.5 ± 12.1		Allied health professionals	547	(9.5)
**Age class**			Medical technician	271	(4.7)
≤30	915	(15.8)	Obstetrician	48	(0.8)
31–49	1869	(32.4)	Pharmacist	36	(0.6)
>50	2042	(35.3)	Psychologist	56	(1.0)
nr	951	(16.5)	Physiotherapist	106	(1.8)
**Country of birth**			Student	251	(4.3)
Italy	5474	(94.8)	Laboratory personnel	159	(2.8)
Other Country UE	108	(1.9)	Administrative personnel	531	(9.2)
Other Country extra UE	115	(2.0)	Other	360	(6.2)
nr	80	(1.4)	nr	234	(4.1)
**Ethnicity**			Prevalent Hospital ward *		
Caucasian	4512	(78.1)	COVID-19 outpatient clinics	129	(2.2)
Hispanic or Latin	428	(7.4)	Non COVID-19 outpatient clinics	1002	(17.3)
African	15	(0.3)	Low-intensity COVID-19 wards	659	(11.4)
Asian	10	(0.2)	High-intensity COVID-19 wards	625	(10.8)
Arabic	8	(0.1)	Non COVID-19 wards	1515	(26.2)
Another Ethnicity	102	(1.8)	Surgical room	545	(9.4)
nr	702	(12.2)	Office	861	(14.9)
**Marital Status**			**No. of SARS-CoV-2 infections**		
Single	1751	(30.3)	0	2978	(51.5)
Married	2562	(44.3)	1	2484	(43.0)
Cohabitant	746	(12.9)	2	276	(4.8)
Divorced	495	(8.6)	3	27	(0.5)
nr	223	(3.9)	nr	12	(0.2)
**Level of education**			**COVID-19 Vaccinations**		
Secondary school diploma	338	(5.9)	yes	5338	(92.4)
High school Diploma	1607	(27.8)	no	24	(0.4)
Degree	2357	(40.8)	nr	415	(7.2)
Postgraduate Education	1250	(21.6)	**Vaccine doses COVID-19**		
nr	225	(3.9)	0	24	(0.4)
**BMI (mean ± SD)**	24.0 ± 4.5		1	17	(0.3)
**BMI**			2	281	(4.9)
Underweight (<18.5)	223	(3.9)	3	5020	(86.9)
Normal Weight (18.5–24.9)	3428	(59.3)	4	17	(0.3)
Overweight (25.0–29.9)	1282	(22.2)	nr	418	(7.2)
Obesity (≥30)	537	(9.3)	**University Hospital**		
nr	307	(5.3)	Modena	255	(4.4)
**Smoker**			Padova	1206	(20.9)
Yes	1549	(26.8)	Trieste	1082	(18.7)
Former	552	(9.6)	Verona	3234	(56.0)
Never	3596	(62.2)			
nr	80	(1.4)			
**At least one comorbidity**	1355	(23.5)			
Diabetes	101	(1.7)			
Cardiovascular	545	(9.4)			
Respiratory chronic	200	(3.5)			
Neurological	123	(2.1)			
Psychiatric	113	(2.0)			
Autoimmune/rheumatological	381	(6.6)			
Chronic kidney	35	(0.6)			
Chronic liver	57	(1.0)			
Immunosuppressive conditions	156	(2.7)			

Legend: nr: subjects who did not respond to the specific question in the online questionnaire. * Prevalent hospital ward at which the subjects worked from February 2020 to March 2022.

**Table 2:** Distribution of the respondents to the questionnaire, based on the number of infections (including at least one infection and reinfections), for the main covariates. The logistic multivariate regression conducted to evaluate the risk of having at least one infection and a reinfection is represented.

	Total	Subjects with at Least One Infection *n* = 2787				Subjects with Reinfections *n* = 303			
	n = 5777	n	(%)	adjOR (95% CI)	*p*	n	(%)	adjOR (95%CI)	*p*
**Sex**									
Female	4349	2129	(49.0)	0.99 (0.86–1.15)	0.920	238	(5.5)	0.94 (0.68–1.3)	0.699
Male	1363	658	(48.3)	ref		65	(4.8)	ref	
**Age class**									
≤30	915	486	(53.1)	**1.64 (1.35–1.99)**	**0.000**	62	(6.8)	**1.94 (1.29–2.93)**	**0.002**
31–49	1869	1014	(54.3)	**1.63 (1.42–1.87)**	**0.000**	99	(5.3)	1.32 (0.96–1.81)	0.084
>50	2042	861	(42.2)	ref		88	(4.3)	ref	
**BMI categories**									
Underweight	223	99	(44.4)	0.83 (0.61–1.12)	0.226	62	(27.8)	0.44 (0.18–1.09)	0.077
Normal Weight	3428	1682	(49.1)	ref		196	(5.7)	ref	
Overweight	1282	642	(50.1)	1.05 (0.91–1.22)	0.519	6	(0.5)	0.73 (0.52–1.03)	0.075
Obesity	537	256	(47.7)	0.98 (0.80–1.21)	0.881	28	(5.2)	0.8 (0.50–1.28)	0.346
**Smoker**									
Yes	1549	764	(49.3)	0.94 (0.82–1.08)	0.361	78	(5.0)	0.82 (0.60–1.12)	0.216
Former	552	267	(48.4)	1.12 (0.91–1.39)	0.276	32	(5.8)	1.31 (0.85–2.03)	0.228
Never	3596	1756	(48.8)	ref		193	(5.4)	ref	
**Job title**									
Physicians	697	334	(47.9)	1.07 (0.86–1.33)	0.567	28	(4.0)	0.98 (0.56–1.71)	0.935
Residents	544	318	(58.5)	1.27 (0.94–1.71)	0.123	32	(5.9)	0.79 (0.38–1.62)	0.514
Nurses	1937	1053	(54.4)	**1.47 (1.24–1.75)**	**0.000**	134	(6.9)	**1.71 (1.13–2.61)**	**0.012**
Allied health professionals	547	270	(49.4)	1.25 (0.99–1.59)	0.064	38	(6.9)	**1.85 (1.09–3.13)**	**0.023**
Other health personnel	768	343	(44.7)	0.94 (0.76–1.17)	0.578	30	(3.9)	0.73 (0.42–1.27)	0.266
Other non-health personnel	1050	442	(42.1)	ref		38	(3.6)	ref	
**Comorbidities**									
Diabetes	101	49	(48.5)	1.27 (0.82–1.97)	0.282	6	(5.9)	1.45 (0.57–3.68)	0.440
Cardiovascular	545	232	(42.6)	0.86 (0.7–1.06)	0.161	24	(4.4)	0.9 (0.54–1.47)	0.663
Respiratory chronic	200	114	(57.0)	**1.57 (1.13–2.18)**	**0.007**	22	(11.0)	**2.19 (1.28–3.72)**	**0.004**
Neurological	123	59	(48.0)	1.03 (0.67–1.58)	0.894	8	(6.5)	1.02 (0.4–2.58)	0.965
Psychiatric	113	62	(54.9)	1.23 (0.81–1.86)	0.341	3	(2.7)	0.40 (0.10–1.66)	0.210
Autoimmune/rheumatological	381	189	(49.6)	1.06 (0.83–1.35)	0.662	23	(6.0)	1.32 (0.81–2.16)	0.264
Chronic kidney	35	23	(65.7)	**2.28 (1.06–4.9)**	**0.035**	2	(5.7)	0.81 (0.11–6.10)	0.837
Chronic liver	57	23	(40.4)	0.8 (0.43–1.52)	0.501	2	(3.5)	0.98 (0.23–4.16)	0.981
Immunosuppressive conditions	156	68	(43.6)	0.85 (0.58–1.23)	0.384	9	(5.8)	1.18 (0.55–2.56)	0.670
**Hospital wards**									
COVID-19 outpatient clinics	129	70	(54.3)	1.07 (0.73–1.57)	0.738	4	(3.1)	0.56 (0.2–1.56)	0.271
COVID-19 inpatient wards	1150	651	(56.6)	**1.17 (1.01–1.37)**	**0.038**	87	(7.6)	**1.43 (1.06–1.93)**	**0.020**
**University Hospital**									
Modena	255	158	(62.0)	**1.68 (1.17–2.4)**	**0.005**	19	(7.5)	**2.13 (1.01–4.48)**	**0.046**
Padova	1206	645	(53.5)	**1.35 (1.16–1.56)**	**0.000**	60	(5.0)	0.96 (0.69–1.34)	0.826
Trieste	1082	560	(51.8)	**1.47 (1.25–1.73)**	**0.000**	68	(6.3)	**1.42 (1.01–2.01)**	**0.042**
Verona	3234	1424	(44.0)	ref		156	(4.8)	ref	

Legend: bold text indicates statistically significant results; ref: reference.

**Table 3:** Distribution of reinfections on the totality of the infections—logistic regression.

	Total *n* = 3117	Reinfections *n* = 330		adjOR (95%CI)	*p*
	n	n.	(%)		
**Hospital ward where infections were contracted**					
Office	374	38	(10.2)	ref	
Non-COVID-19 outpatient clinics	409	38	(9.3)	1.03 (0.63–1.67)	0.909
COVID-19 outpatient clinics	41	7	(17.1)	2.22 (0.87–5.64)	0.094
Non-COVID-19 hospital ward	751	89	(11.9)	1.39 (0.92–2.11)	0.120
Low-intensity COVID-19 wards	209	26	(12.4)	1.63 (0.94–2.84)	0.083
High-intensity COVID-19 wards	166	26	(15.7)	**1.91 (1.09–3.35)**	**0.023**
Surgical room	138	11	(8.0)	0.91 (0.44–1.87)	0.799
Others	1029	95	(9.2)	1 (0.66–1.5)	0.990
**Phase in which infections occurred**					
Phase 1	167	0	(0.0)		
Phase 2	431	12	(2.8)	ref	
Phase 3	109	9	(8.3)	**6.04 (2.15–16.92)**	**0.001**
Phase 4	899	88	(9.8)	**16.8 (6.74–41.87)**	**0.000**
Phase 5	1024	129	(12.6)	**29.44 (11.77–73.68)**	**0.000**
**Vaccination status for positive cases**					
0 doses	540	21	(3.9)	ref	
1 doses	45	8	(17.8)	0.75 (0.26–2.17)	0.596
2 doses	230	57	(24.8)	0.83 (0.38–1.81)	0.632
3 doses	1366	107	(7.8)	**0.17 (0.08–0.36)**	**0.000**

Legend: bold text indicates statistically significant results; ref: reference.

**Table 4:** Infection outcomes—First infections versus reinfection, bivariate analysis.

	First Infections *n* = 2787		Reinfection *n* = 330		Total *n* = 3117		*p*
	*n*	(%)	*n*	(%)	*n*	(%)	
**Duration of positivity (days)**	14.0 ± 8.4		10.1 ± 5.0		13.6 ± 8.3		**<0.001**
**Symptoms during positivity**							
yes	1855	(66.6)	150	(45.5)	2005	(64.3)	**<0.001**
no/nr	932	(33.4)	180	(54.5)	1112	(35.7)	
**Duration of symptoms (days)**	6.4 ± 6.4		4.6 ± 2.8		6.2 ± 6.2		**<0.001**
**Hospitalization**							
yes	18	(0.6)	1	(0.3)	19	(0.6)	0.449
no/nr	2769	(99.4)	329	(99.7)	3098	(99.4)	
Duration of hospitalization (days) *	8.8 ± 6.5		1		8.5 ± 6.6		0.272
**ICU admission**							
yes	4	(0.1)	0	(0.0)	4	(0.1)	0.491
no/nr	2783	(99.9)	330	(100.0)	3113	(99.9)	
**Symptoms after negative swab**							
yes	1296	(46.5)	98	(29.7)	1394	(44.7)	**<0.001**
no/nr	1491	(53.5)	232	(70.3)	1723	(55.3)	
**Duration of symptoms after negative swab**							
Less than 15 days	297	(22.9)	21	(21.4)	318	(22.8)	0.236
16–30 days	298	(23.0)	24	(24.5)	322	(23.1)	
30–60 days	193	(14.9)	12	(12.2)	205	(14.7)	
More than 60 days	189	(14.6)	8	(8.2)	197	(14.1)	
Still present	283	(21.8)	28	(28.6)	311	(22.3)	
nr	36	(2.8)	5	(5.1)	41	(2.9)	
**Follow up of outcomes after negative swab**							
yes	138	(5.0)	17	(5.2)	155	(5.0)	0.874
no/nr	2649	(95.0)	313	(94.8)	2962	(95.0)	

Legend: bold text indicates statistically significant results. nr: subjects who did not respond to the specific question in the online questionnaire. “ICU”: intensive care unit. * Duration of the hospitalization calculated exclusively for the HCWs hospitalized.

**Table 5:** Distribution of infections with symptoms during positivity and persistent symptoms after a negative SARS-CoV-2 swab—a logistic regression analysis.

	Total Infections	Infections with Symptoms During Positivity *n* = 2005				Infections with Persistent Symptoms After Negative SARS-CoV-2 Swab *n* = 1394			
	*n* = 3117	*n*.	(%)	adjOR (95%CI)	*p*	*n*	(%)	adjOR (95%CI)	*p*
**Sex**									
Female	2385	1589	(66.6)	**1.49 (1.25–1.79)**	**0.000**	1162	(48.7)	**1.99 (1.66–2.38)**	**0.000**
Male	732	416	(56.8)	ref		232	(31.7)	ref	
**Age class**									
≤30	550	398	(72.4)	**1.46 (1.14–1.86)**	**0.002**	197	(35.8)	**0.62 (0.49–0.77)**	**0.000**
31–49	1120	717	(64.0)	0.94 (0.78–1.14)	0.562	497	(44.4)	0.84 (0.70–1.01)	0.065
>50	957	616	(64.4)	ref		484	(50.6)	ref	
**Comorbidities**									
yes	738	460	(62.3)	0.94 (0.78–1.13)	0.538	396	(53.7)	**1.50 (1.26–1.78)**	**0.000**
no	2379	1545	(64.9)	ref		998	(42.0)	ref	
**Reinfection**									
yes	330	150	(45.5)	**0.42 (0.33–0.54)**	**0.000**	98	(29.7)	**0.54 (0.42–0.7)**	**0.000**
no	2787	1855	(66.6)	ref		1296	(46.5)	ref	
**Study phase**									
1	167	87	(52.1)	ref		91	(54.5)	ref	
2	431	304	(70.5)	**2.16 (1.49–3.14)**	**0.000**	249	(57.8)	1.07 (0.74–1.55)	0.718
3	109	55	(50.5)	1.20 (0.69–2.11)	0.518	52	(47.7)	0.72 (0.41–1.26)	0.248
4	899	601	(66.9)	**2.52 (1.55–4.09)**	**0.000**	370	(41.2)	**0.51 (0.31–0.82)**	**0.005**
5	1024	772	(75.4)	**3.97 (2.42–6.51)**	**0.000**	458	(44.7)	**0.59 (0.36–0.95)**	**0.031**
**Vaccination status** **during positivity**									
0	540	356	(65.9)	ref		302	(55.9)	ref	
1	45	30	(66.7)	1.08 (0.51–2.28)	0.834	23	(51.1)	1.46 (0.72–2.95)	0.290
2	230	135	(58.7)	**0.58 (0.36–0.93)**	**0.025**	93	(40.4)	1.03 (0.64–1.64)	0.916
3	1366	996	(72.9)	0.75 (0.49–1.16)	0.192	620	(45.4)	1.26 (0.83–1.91)	0.281

Legend: bold text indicates statistically significant results. nr: subjects who did not respond to the specific question in the online questionnaire; ref: reference.

**Table 6:** Duration of positivity. Linear regression analysis (Y = duration of positivity, in days).

	Mean Duration of Positivity 13.6 ± 8.3 days	Beta Coefficient	*p*	(95% CI)
**Sex (female vs. male)**		−0.24	0.529	(−1.00; 0.51)
Male	13.7			
Female	13.6			
**Age**		0.04	**0.003**	(0.01; 0.07)
**Comorbidities (yes vs. no)**				
yes	14.3	0.15	0.689	(−0.60; 0.91)
no	13.4			
**Reinfection (yes vs. no)**				
yes	10.1	−1.93	**0.001**	(−3.05; −0.80)
no	14.0			
**Vaccination (yes vs. no)**				
yes	11.1	−3.61	**0.000**	(−5.15; −2.08)
no	21.5			
**Study phase**		−2.50	**0.000**	(−3.02; −1.98)
1	25.1			
2	20.5			
3	14.9			
4	12.2			
5	10.1			

**Table 7:** Duration of symptoms. Linear regression analysis (Y = duration of symptoms, in days).

	Mean Duration of Symptoms 6.2 ± 6.2 days	Beta Coefficient	*p*	(95%CI)
**Sex (female vs. male)**		1.46	**0.001**	(0.63; 2.29)
Male	4.9			
Female	6.6			
**Age**		0.06	**0.000**	(0.03; 0.09)
**Comorbidities (yes vs. no)**				
yes	7.5	0.86	**0.041**	(0.03; 1.68)
no	5.9			
**Reinfection (yes vs. no)**				
yes	4.6	−0.61	0.353	(−1.91; 0.68)
no	6.4			
**Vaccination (yes vs. no)**				
yes	5.1	−2.27	**0.013**	(−4.03; −0.49)
no	10.3			
**Study phase**		−0.95	**0.002**	(−1.55; −0.35)
1	10.2			
2	10.5			
3	6.4			
4	5.6			
5	4.7			

## Data Availability

The datasets generated during the current study are not publicly available because they contain sensitive data to be treated under data protection laws and regulations. Appropriate forms of data sharing can be arranged after a reasonable request to the first author.

## Supplementary Materials

The following supporting information can be downloaded at https://www.mdpi.com/article/10.3390/vaccines13080815/s1: Table S1: Distribution of symptoms among symptomatic infections (during positivity)—for first infections versus reinfections—based on bivariate analysis; Table S2: Distribution of symptoms among infections with persistent symptoms (after a negative SARS-CoV-2 swab)—for first infections versus reinfections—based on bivariate analysis.; Table S3: Distribution of COVID-19-related symptoms based on logistic multivariate analysis.; Table S4: Distribution of COVID-19-related symptoms based on logistic multivariate analysis.; Table S5: Distribution of COVID-19-related symptoms based on logistic multivariate analysis.

## Abbreviations

The following abbreviations are used in this manuscript:

SARS-CoV-2	Severe Acute Respiratory Syndrome Coronavirus 2
COVID-19	Coronavirus Disease 2019
HCWs	Healthcare Workers
RT-PCR	Reverse Transcription-Polymerase Chain Reaction
ICU	Intensive Care Unit
BMI	Body Mass Index
adjOR	Adjusted Odds Ratio
CI	Confidence Interval
BI	Breakthrough Infection
SPSS	Statistical Package for the Social Sciences
ASUGI	Azienda Sanitaria Universitaria Giuliano-Isontina
ORCHESTRA	Connecting European Cohorts to Increase Common and Effective Response to SARS-CoV-2 Pandemic
