# Supplementary material for: Risk of SARS-CoV-2 Reinfections Among Healthcare Workers of Four Large University Hospitals in Northern Italy: Results of an Online Survey Within the ORCHESTRA Project
Source: Vaccines (Basel). 2025 Jul 31;13(8):815. doi: 10.3390/vaccines13080815 (PMC12390568; doi:10.3390/vaccines13080815)
Supplement: Supplementary file 1 [file vaccines-13-00815-s001.zip › vaccines-3695868-supplementary.pdf]

Supplementary Materials

Table S1. Distribution of symptoms among symptomatic infections (during positivity) - First infections versus reinfections - Bivariate Analysis.

Symptoms during positivity	First infections n=1,855		Reinfections n=150		Total symptomatic infections n=2,005		OR (95%CI)	p
	n	(%)	n	(%)	n	(%)		
Fever	1,078	(58.1)	72	(48.0)	1,150	(57.4)	**0.67 (0.48 - 0.93)**	**0.016**
Fatigue and Malaise	1,394	(75.1)	89	(59.3)	1,483	(74.0)	**0.48 (0.34 - 0.68)**	**<0.001**
Myalgia-Arthralgia	1,079	(58.2)	69	(46.0)	1,148	(57.3)	**0.61 (0.44 - 0.86)**	**0.004**
Cough	1,015	(54.7)	69	(46.0)	1,084	(54.1)	**0.71 (0.51 - 0.98)**	**0.041**
Headache	983	(53.0)	65	(43.3)	1,048	(52.3)	**0.68 (0.49 - 0.95)**	**0.023**
Runny Nose	841	(45.3)	61	(40.7)	902	(45.0)		ns
Sore Throat	800	(43.1)	68	(45.3)	868	(43.3)		ns
Chills	592	(31.9)	41	(27.3)	633	(31.6)		ns
Loss of Taste	507	(27.3)	18	(12.0)	525	(26.2)	**0.36 (0.22 - 0.60)**	**<0.001**
Loss of Smell	486	(26.2)	15	(10.0)	501	(25.0)	**0.31 (0.18 - 0.54)**	**<0.001**
Rhinitis	532	(28.7)	42	(28.0)	574	(28.6)		ns
Dyspnoea	400	(21.6)	19	(12.7)	419	(20.9)	**0.53 (0.32 - 0.86)**	**0.010**
Lack of Appetite	278	(15.0)	9	(6.0)	287	(14.3)	**0.36 (0.18 - 0.72)**	**0.003**
Difficulty Concentrating	248	(13.4)	15	(10.0)	263	(13.1)		ns
Diarrhea	234	(12.6)	16	(10.7)	250	(12.5)		ns
Chest Pain	204	(11.0)	10	(6.7)	214	(10.7)		ns
Insomnia	212	(11.4)	13	(8.7)	225	(11.2)		ns
Anxious State	204	(11.0)	9	(6.0)	213	(10.6)		ns
Weight Loss	153	(8.2)	8	(5.3)	161	(8.0)		ns
Abdominal Pain	121	(6.5)	9	(6.0)	130	(6.5)		ns
Vomiting and Nausea	179	(9.6)	7	(4.7)	186	(9.3)	**0.46 (0.21 - 0.99)**	**0.041**
Conjunctivitis	103	(5.6)	4	(2.7)	107	(5.3)		ns
Skin Lesions	31	(1.7)	2	(1.3)	33	(1.6)		ns
Lymphadenopathy	50	(2.7)	1	(0.7)	51	(2.5)		ns
Bleeding	6	(0.3)	0	(0.0)	6	(0.3)		ns
Confusion	59	(3.2)	2	(1.3)	61	(3.0)		ns
Aphasia	18	(1.0)	0	(0.0)	18	(0.9)		ns
Memory Loss	87	(4.7)	9	(6.0)	96	(4.8)		ns
Dizziness	93	(5.0)	6	(4.0)	99	(4.9)		ns
Seizures	2	(0.1)	0	(0.0)	2	(0.1)		ns
Inability to Walk	31	(1.7)	2	(1.3)	33	(1.6)		ns
Fainting	22	(1.2)	0	(0.0)	22	(1.1)		ns
Depression	85	(4.6)	5	(3.3)	90	(4.5)		ns

Legend: bold indicates statistically significant results; ns: not significative association

Table S2. Distribution of symptoms among infections with persistent symptoms (after the negative SARS-CoV-2 swab) - First infections versus reinfections - Bivariate analysis.

Symptoms after negative SARS-CoV-2 swab	First infections n=1,296		Reinfections n=98		Total symptomatic infections n=1,394		p
	n	(%)	n	(%)	n	(%)	
Fatigue and Malaise	644	(49.7)	45	(45.9)	689	(49.4)	ns
Myalgia-Arthralgia	286	(22.1)	30	(30.6)	316	(22.7)	ns
Cough	267	(20.6)	19	(19.4)	286	(20.5)	ns
Dyspnoea	215	(16.6)	17	(17.3)	232	(16.6)	ns
Difficulty Concentrating	177	(13.7)	18	(18.4)	195	(14.0)	ns
Headache	157	(12.1)	13	(13.3)	170	(12.2)	ns
Loss of Taste	127	(9.8)	4	(4.1)	131	(9.4)	ns
Loss of Smell	141	(10.9)	7	(7.1)	148	(10.6)	ns
Runny Nose	93	(7.2)	9	(9.2)	102	(7.3)	ns
Rhinitis	83	(6.4)	3	(3.1)	86	(6.2)	ns
Insomnia	107	(8.3)	12	(12.2)	119	(8.5)	ns
Anxious State	86	(6.6)	6	(6.1)	92	(6.6)	ns
Fever	10	(0.8)	0	(0.0)	10	(0.7)	ns
Chills	9	(0.7)	0	(0.0)	9	(0.6)	ns
Weight Loss	21	(1.6)	0	(0.0)	21	(1.5)	ns
Abdominal Pain	18	(1.4)	0	(0.0)	18	(1.3)	ns
Vomiting and Nausea	22	(1.7)	3	(3.1)	25	(1.8)	ns
Diarrhea	26	(2.0)	2	(2.0)	28	(2.0)	ns
Chest Pain	40	(3.1)	2	(2.0)	42	(3.0)	ns
Sore Throat	46	(3.5)	7	(7.1)	53	(3.8)	ns
Conjunctivitis	14	(1.1)	1	(1.0)	15	(1.1)	ns
Skin Lesions	9	(0.7)	0	(0.0)	9	(0.6)	ns
Lymphadenopathy	5	(0.4)	1	(1.0)	6	(0.4)	ns
Bleeding	1	(0.1)	0	(0.0)	1	(0.1)	ns
Confusion	27	(2.1)	1	(1.0)	28	(2.0)	ns
Aphasia	5	(0.4)	0	(0.0)	5	(0.4)	ns
Memory Loss	77	(5.9)	8	(8.2)	85	(6.1)	ns
Dizziness	23	(1.8)	2	(2.0)	25	(1.8)	ns
Seizures	1	(0.1)	0	(0.0)	1	(0.1)	ns
Inability to Walk	6	(0.5)	1	(1.0)	7	(0.5)	ns
Fainting	3	(0.2)	0	(0.0)	3	(0.2)	ns
Lack of Appetite	20	(1.5)	0	(0.0)	20	(1.4)	ns
Depression	40	(3.1)	4	(4.1)	44	(3.2)	ns

Legend: ns: not significative association.

Table S3. Distribution of COVID-19-related symptoms - Logistic Multivariate Analysis.

	Symptomatic infections	Fever n=1,150				Fatigue/Malaise n=1,483			
	**n.2,005**	**n.**	**(%)**	**adjOR (95%CI)**	**p**	**n.**	**(%)**	**adjOR (95%CI)**	**p**
**Sex**									
Female	1,589	886	(55.8)	**0.76 (0.61 - 0.96)**	**0.020**	1,202	(75.6)	**1.5 (1.18 - 1.91)**	**0.001**
Male	416	264	(63.5)	ref		281	(67.5)	ref	
**Age class**									
≤30	398	240	(60.3)		ns	295	(74.1)		ns
31-49	717	408	(56.9)			527	(73.5)		
>50	616	343	(55.7)			472	(76.6)		
nr	274	159	(58.0)			189	(69.0)		
**Comorbidities**									
yes	460	273	(59.3)		ns	360	(78.3)	**1.33 (1.03 - 1.71)**	**0.031**
no	1,545	877	(56.8)			1,123	(72.7)		
**Reinfection**									
yes	150	72	(48.0)	**0.62 (0.44 - 0.88)**	**0.007**	89	(59.3)	**0.51 (0.36 - 0.73)**	**0.000**
no	1,855	1,078	(58.1)	ref		1,394	(75.1)	ref	
**Study phase**									
1	87	61	(70.1)	ref		72	(82.8)		ns
2	304	178	(58.6)	0.61 (0.37 - 1.03)	0.064	251	(82.6)		
3	55	28	(50.9)	**0.43 (0.20 - 0.93)**	**0.031**	42	(76.4)		
4	601	296	(49.3)	**0.48 (0.25 - 0.92)**	**0.027**	430	(71.5)		
5	772	483	(62.6)	0.86 (0.44 - 1.65)	0.642	558	(72.3)		
nr	186	104	(55.9)			130	(69.9)		
**Vaccination status to the positivity**									
0	356	216	(60.7)		ns	291	(81.7)		ns
1	30	20	(66.7)			23	(76.7)		
2	135	78	(57.8)			95	(70.4)		
3	996	560	(56.2)			721	(72.4)		
nr	488	276	(56.6)			353	(72.3)		

	Symptomatic infections	Myalgia/arthralgia n=1,148				Cough n=1,084			
	**n=2,005**	**n.**	**(%)**	**adjOR (95%CI)**	**p**	**n.**	**(%)**	**adjOR (95%CI)**	**p**
**Sex**									
Female	1,589	951	(59.8)	**1.63 (1.31 - 2.04)**	**0.000**	860	(54.1)		ns
Male	416	197	(47.4)	ref		224	(53.8)		
**Age class**									
≤30	398	210	(52.8)		ns	247	(62.1)	**1.56 (1.19 - 2.05)**	**0.001**
31-49	717	399	(55.6)			375	(52.3)	1.07 (0.86 - 1.34)	0.548
>50	616	382	(62.0)			316	(51.3)	ref	
nr	274	157	(57.3)			146	(53.3)		
**Comorbidities**									
yes	460	288	(62.6)		ns	260	(56.5)		ns
no	1,545	860	(55.7)			824	(53.3)		
**Reinfection**									
yes	150	69		**0.66 (0.47 - 0.93)**	**0.018**	69	(46.0)	**0.56 (0.40 - 0.80)**	**0.001**
no	1,855	1,079	(58.2)	ref		1,015	(54.7)	ref	
**Study phase**									
1	87	57	(65.5)		ns	35	(40.2)	**0.24 (0.12 - 0.46)**	**0.000**
2	304	218	(71.7)			121	(39.8)	**0.22 (0.13 - 0.39)**	**0.000**
3	55	27	(49.1)			29	(52.7)	**0.53 (0.29 - 0.97)**	**0.038**
4	601	314	(52.2)			293	(48.8)	**0.49 (0.40 - 0.62)**	**0.000**
5	772	432	(56.0)			505	(65.4)	ref	
nr	186	100	(53.8)			101	(54.3)		
**Vaccination status to the positivity**									
0	356	245	(68.8)		ns	150	(42.1)	1.60 (0.92 - 2.80)	0.097
1	30	14	(46.7)			7	(23.3)	**0.35 (0.14 - 0.84)**	**0.019**
2	135	74	(54.8)			76	(56.3)	1.21 (0.83 - 1.78)	0.324
3	996	541	(54.3)			580	(58.2)	ref	
nr	488	274	(56.1)			271	(55.5)		

Legend: bold indicates statistically significant results; ns: not significative association; ref: reference; nr: subjects who did not respond to the specific question in the online questionnaire.

Table S4. Distribution of COVID-19-related symptoms - Logistic multivariate analysis.

	Symptomatic infections	Headache n=1,048				Vomiting n=186			
	n=2,005	n.	(%)	adjOR (95%CI)	p	n.	(%)	adjOR (95%CI)	p
**Sex**									
Female	1,589	885	(55.7)	**1.94 (1.55 - 2.42)**	**0.000**	173	(10.9)	**3.93 (2.2 - 7.03)**	**0.000**
Male	416	163	(39.2)	Ref		13	(3.1)	ref	
**Age class**									
≤30	398	196	(49.2)		ns	32	(8.0)		ns
31-49	717	386	(53.8)			62	(8.6)		
>50	616	330	(53.6)			73	(11.9)		
nr	274	136	(49.6)			19	(6.9)		
**Comorbidities**									
yes	460	251	(54.6)		ns	49	(10.7)		ns
no	1,545	797	(51.6)			137	(8.9)		
**Reinfection**									
yes	150	65	(43.3)	**0.67 (0.48 - 0.95)**	**0.026**	7	(4.7)	0.47 (0.21 - 1.04)	0.061
no	1,855	983	(53.0)	ref		179	(9.6)	ref	
**Study phase**									
1	87	46	(52.9)		ns	14	(16.1)	ref	
2	304	175	(57.6)			42	(13.8)	0.71 (0.36 - 1.39)	0.313
3	55	30	(54.5)			7	(12.7)	0.62 (0.20 - 1.93)	0.410
4	601	313	(52.1)			35	(5.8)	**0.30 (0.11 - 0.83)**	**0.020**
5	772	397	(51.4)			69	(8.9)	0.54 (0.20 - 1.44)	0.218
nr	186	87	(46.8)			19	(10.2)		
**Vaccination status to the positivity**									
0	356	200	(56.2)		ns	51	(14.3)		ns
1	30	17	(56.7)			3	(10.0)		
2	135	72	(53.3)			13	(9.6)		
3	996	517	(51.9)			75	(7.5)		
nr	488	242	(49.6)			44	(9.0)		

	Symptomatic infections	Dyspnoea n=419				Loss of appetite n=287			
	n=2,005	n.	(%)	adjOR (95%CI)	p	n.	(%)	adjOR (95%CI)	p
**Sex**									
Female	1,589	370	(23.3)	**2.24 (1.61 - 3.11)**	**0.000**	249	(15.7)	**1.94 (1.34 - 2.81)**	**0.000**
Male	416	49	(11.8)	ref		38	(9.1)	ref	
**Age class**									
≤30	398	71	(17.8)		ns	60	(15.1)		ns
31-49	717	129	(18.0)			94	(13.1)		
>50	616	155	(25.2)			99	(16.1)		
nr	274	64	(23.4)			34	(12.4)		
**Comorbidities**									
yes	460	137	(29.8)	**1.82 (1.41 - 2.34)**	**0.000**	68	(14.8)		ns
no	1,545	282	(18.3)			219	(14.2)		
**Reinfection**									
yes	150	19	(12.7)		ns	9	(6.0)	**0.39 (0.19 - 0.80)**	**0.010**
no	1,855	400	(21.6)			278	(15.0)	ref	
**Study phase**									
1	87	30	(34.5)	**2.98 (1.45 - 6.10)**	**0.003**	26	(29.9)	**2.40 (1.12 - 5.16)**	**0.025**
2	304	113	(37.2)	**3.06 (1.66 - 5.63)**	**0.000**	70	(23.0)	1.54 (0.79 - 3.03)	0.207
3	55	10	(18.2)	1.13 (0.52 - 2.44)	0.757	10	(18.2)	1.10 (0.50 - 2.41)	0.820
4	601	112	(18.6)	1.25 (0.93 - 1.68)	0.136	48	(8.0)	**0.45 (0.31 - 0.65)**	**0.000**
5	772	116	(15.0)	ref		119	(15.4)	ref	
nr	186	38	(20.4)			14	(7.5)		
**Vaccination status to the positivity**									
0	356	124	(34.8)		ns	84	(23.6)		ns
1	30	6	(20.0)			1	(3.3)		
2	135	24	(17.8)			18	(13.3)		
3	996	160	(16.1)			122	(12.2)		
nr	488	105	(21.5)			62	(12.7)		

Legend: bold indicates statistically significant results; ns: not significative association; ref: reference; nr: subjects who did not respond to the specific question in the online questionnaire.

Table S5. Distribution of COVID-19-related symptoms - Logistic multivariate analysis.

	Symptomatic infections n=2,005	Loss of taste n=525				Loss of smell n=501			
		**n.**	**(%)**	**adjOR (95%CI)**	**p**	**n.**	**(%)**	**adjOR (95%CI)**	**p**
**Sex**									
Female	1,589	437	(27.5)	**1.40 (1.06 - 1.85)**	**0.018**	406	(25.6)		ns
Male	416	88	(21.2)	ref		95	(22.8)	ref	
**Age class**									
≤30	398	84	(21.1)		ns	91	(22.9)		ns
31-49	717	186	(25.9)			179	(25.0)		
>50	616	178	(28.9)			154	(25.0)		
nr	274	77	(28.1)			77	(28.1)		
**Comorbidities**									
yes	460	131	(28.5)		ns	115	(25.0)		ns
no	1,545	394	(25.5)			386	(25.0)		
**Reinfection**									
yes	150	18	(12.0)	**0.50 (0.30 - 0.84)**	**0.009**	15	(10.0)	**0.43 (0.25 - 0.76)**	**0.004**
no	1,855	507	(27.3)	ref		486	(26.2)	ref	
**Study phase**									
1	87	49	(56.3)	**10.38 (5.12 - 21.01)**	**0.000**	53	(60.9)	**9.89 (4.90 - 19.96)**	**0.000**
2	304	166	(54.6)	**9.42 (5.10 - 17.41)**	**0.000**	171	(56.3)	**8.05 (4.39 - 14.78)**	**0.000**
3	55	22	(40.0)	**3.53 (1.84 - 6.77)**	**0.000**	21	(38.2)	**3.07 (1.59 - 5.92)**	**0.001**
4	601	110	(18.3)	**1.08 (0.81 - 1.44)**	**0.610**	101	(16.8)	**1.14 (0.85 - 1.55)**	**0.383**
5	772	127	(16.5)	ref		108	(14.0)	ref	
nr	186	51	(27.4)			47	(25.3)		
**Vaccination status to the positivity**									
0	356	186	(52.2)		ns	198	(55.6)	0.97 (0.52 - 1.79)	0.916
1	30	8	(26.7)			8	(26.7)	1.45 (0.59 - 3.59)	0.417
2	135	36	(26.7)			36	(26.7)	**1.95 (1.24 - 3.06)**	**0.004**
3	996	176	(17.7)			147	(14.8)	ref	
nr	488	119	(24.4)			112	(23.0)		

Legend: bold indicates statistically significant results; ns: not significative association; ref: reference; nr: subjects who did not respond to the specific question in the online questionnaire.
